# Effect of Grape Pomace Polyphenols With or Without Pectin on TMAO Serum Levels Assessed by LC/MS-Based Assay: A Preliminary Clinical Study on Overweight/Obese Subjects

**DOI:** 10.3389/fphar.2019.00575

**Published:** 2019-05-21

**Authors:** Giuseppe Annunziata, Maria Maisto, Connie Schisano, Roberto Ciampaglia, Viviana Narciso, Sherif T. S. Hassan, Gian Carlo Tenore, Ettore Novellino

**Affiliations:** ^1^Department of Pharmacy, University of Naples Federico II, Naples, Italy; ^2^Department of Natural Drugs, Faculty of Pharmacy, University of Veterinary and Pharmaceutical Sciences Brno, Brno, Czechia

**Keywords:** TMAO, LC-MS, mass spectrometry, polyphenols, grape pomace, nutraceutical, clinical study

## Abstract

Growing evidence suggests that trimethylamine N-oxide (TMAO) is recognized as a biomarker of increased cardiovascular risk. So far, the evaluation of TMAO serum levels in the clinical practice is limited due to the lack of developing new facile methods with reduced limitations. However, few approaches were achieved to determine TMAO in serum by using mass spectrometry-based technique, some limitations were reported including the use of internal standards. Therefore, in this work, a liquid chromatography-mass spectrometry (LC/MS) based-assay was developed to evaluate the effect of grape pomace extract (Taurisolo^®^, group A) or Taurisolo^®^+pectin (group B) on TMAO serum levels in a cohort of overweight/obese subjects. The serum levels of TMAO have been assessed before and after treatment, through LC/MS analysis. After 8-week treatment, in both intervention groups TMAO serum levels significantly decreased (-78.58% *p* = 0.006 and -76.76% *p* = 0.001, group A and group B, respectively). Moreover, we performed several analyses aimed to validate the LC/MS method we used. The method has high precision (% C.V = from 12.12 to 3.92% and from 8.25 to 1.07% for intraday and interday, respectively) and accuracy (% bias = from -5.52 to 0.5% and from -1.42 to 3.08% for intraday and interday, respectively). TMAO recoveries from serum ranged from 99 to 97%; LOD: 2 ng/ml and LOQ: 6 ng/ml. In conclusion, we demonstrated the efficacy of a novel nutraceutical formulation in reducing TMAO serum levels in high cardiovascular risk-subjects, and proposed a useful, versatile and rapid LC/MS method for identification and quantization of TMAO, without the use of marked/isotopic internal standards. It, thus, may represent a novel and practical method with applications in clinical practice and nutraceutical research.

**Clinical Trial Registration:** This study is listed on the ISRCTN registry with ID ISRCTN10794277 (doi: 10.1186/ISRCTN10794277).

## Introduction

Trimethylamine N-oxide (TMAO) has been currently recognized as a prognostic marker for cardiovascular events beyond traditional risk factors (Li et al., 2017). Several studies indicated TMAO as a risk factor for a number of cardiovascular diseases (CVD), including atherosclerosis (Randrianarisoa et al., 2016), stroke (Haghikia et al., 2018; Liang et al., 2018; Nie et al., 2018; Wu et al., 2018), heart failure (Tang et al., 2014). Interestingly, TMAO has been recently found to be positively associated with both visceral Adiposity Index (VAI) and Fatty Liver Index (FLI), that are recognized as a gender-specific indicator of adipose dysfunction and a predictor of non-alcoholic fatty liver disease (NAFLD), respectively (Barrea et al., 2018a). Chemically, TMAO has a structural formula (CH_3_)_3_NO (Ufnal et al., 2015; Subramaniam and Fletcher, 2018) and it mainly derives from the gut microbiota metabolism (Subramaniam and Fletcher, 2018). Selected gut bacteria metabolize various food-derived compounds, such as choline and L-carnitine (Subramaniam and Fletcher, 2018), producing trimethylamine (TMA), that is oxidized in the liver by flavin-containing monooxygenase-3 (FMO3) (Wang et al., 2011; Bennett et al., 2013; Koeth et al., 2014).

Several methods have been developed for the determination of TMAO in biological fluids, such as serum and urine, including nuclear magnetic resonance spectroscopy (NMR) (Murphy et al., 2000; Podadera et al., 2005; Lam et al., 2014), gas chromatography (GC) (Zhang et al., 1995; Mills et al., 1999), high-performance liquid chromatography (HPLC) (Cháfer-Pericás et al., 2004) and mass spectrometry (MS) (Mamer et al., 1999; Hsu et al., 2007; Zhao et al., 2015). MS and NMR are emerging as the most promising methods for the detection of a large body of metabolites, including TMAO (Cheng et al., 2017; Albert and Tang, 2018). The two approaches are different, and pros and cons should be identified. In NMR method, samples can be minimally processed (Cheng et al., 2017) and metabolites detection and quantification are based on chemical shifts in resonance frequency when the compounds are undergoing electromagnetic field (Tenori et al., 2013; Deidda et al., 2015). However, NMR approach has a low sensitivity and it is generally used for the study of the most present of metabolites in the sample (Cheng et al., 2017). By the contrary, MS approach is based on the identification of compounds detecting their unique mass/charge (*m*/*z*) ratio (Albert and Tang, 2018). Although the sample preparation is more complex, requiring the extraction of the compounds from the matrix, MS methods can be used either in an “untargeted” or “targeted” fashion, allowing for the study of a large number of compounds (Cheng et al., 2017; Albert and Tang, 2018). Additionally, MS approaches are more sensitive and routine than NMR (Cheng et al., 2017; Albert and Tang, 2018), thus they may be more commonly used also in clinical diagnostics.

In a previous study, three phosphatidylcholine metabolites were identified: betaine (*m*/*z* 116), choline (*m*/*z* 104), and TMAO (*m*/*z* 76) (Wang et al., 2011). Furthermore, Le et al. (2018) proposed other MS-based methods for detection of TMAO in biofluids, but the use of marked/isotopic internal standards avoided their use in practical applications. In the present study, we monitored the serum TMAO levels by MS-based assay in a cohort of overweight/obese subjects participating to a clinical trial aimed to evaluate the effect of grape pomace extract (registered as Taurisolo^®^) or Taurisolo^®^+pectin on the blood level of this metabolite. We also evaluated the effect of our nutraceutical formulation on oxidized low-density lipoprotein (ox-LDL), as an oxidative stress circulating biomarker. Additionally, we validated the MS-based method for detection and quantification of TMAO in serum without the use of marked/isotopic internal standard.

## Materials and Methods

### Reagents

All chemicals, standards and reagents used were either analytical and mass grade reagents. The water was treated in a Milli-Q water purification system (Millipore, Bedford, MA, United States) before use.

### Nutraceutical Preparation and Formulation

Taurisolo^®^ is a grape pomace polyphenolic extract microencapsulated with maltodextrins, obtained from Aglianico cultivar grape, collected during the harvest in the autumn 2016. The nutraceutical formulations herein used consisted of acid-resistant capsules containing Taurisolo^®^ (300 mg per capsule) or Taurisolo^®^+pectin (300 mg+300 mg per capsule). The extract was encapsulated in acid-resistant capsules, according to our previous studies of polyphenol bioaccessibility (Annunziata et al., 2018, 2019). The supplement was formulated by our Department and the Taurisolo^®^ large scale production has been realized by MBMed Company (Turin, Italy). Briefly, grape has been subjected to ethanolic extraction processes, and sugar has been removed keeping the extract at -20°C for 24 h. The microencapsulation with maltodextrin has been performed through a spray-drying process.

### Study Design, Setting, and Population

Study participants were overweight/obese subjects aged from 18 to 83 years, enrolled in September 2018. Smokers and subjects with hepatic disease, renal disease, heart disease, family history of chronic diseases, in drug therapy or supplement intake containing grape polyphenols, practicing heavy physical exercise (over 10 h per week), pregnant, suspected of being pregnant or hoping to become pregnant, breastfeeding, birch pollen allergy, using vitamin or mineral supplements 2 weeks prior to entry into the study and donating of blood less than 3 months prior to the study were excluded. All subjects gave their informed consent for inclusion before they participated in the study. The study was conducted in accordance with the Declaration of Helsinki, and the protocol was approved by the Ethics Committee of AO Rummo Hospital (Benevento, Italy) (protocol 106 no. 123512 of 18/06/2018). This study is listed on the ISRCTN registry (www.isrctn.com) with ID ISRCTN10794277 (doi: 10.1186/ISRCTN10794277). This study was designed as a 16-week monocentric, double-blind, randomized, placebo-controlled, 2-arm parallel-group trial. Subjects were randomly allocated in two intervention groups: group A (300 mg Taurisolo^®^ twice daily) and group B (300 mg Taurisolo^®^+300 mg pectin twice daily). The study consisted of 4-week run-in period, 8-week intervention period and a 4-week follow-up period. During the run-in period, subjects were given placebo (maltodextrins). Participants were asked to maintain their usual lifestyle habits throughout the entire study duration. Standardized and periodic telephone interviews were performed by qualified personnel in order to verify and increase the protocol compliance, and self-administered life quality questionnaires were completed by subjects during the clinic visits. Blood samples were collected after 12 h of fasting at weeks 0, 4, 8, 12, and 16 in 10-mL EDTA-coated tubes (Becton Dickinson, Plymouth, United Kingdom) and plasma was isolated by centrifugation (20 min, 2,200 *g*, 4°C). All samples were stored at -80°C until analysis. Subjects were asked to abstain from alcohol consumption and practice of hard physical activity 48 h prior to blood sampling.

### Evaluation of the Oxidized Low-Density Lipoprotein Levels

The plasma levels of ox-LDL were measured by using the LP-CHOLOX test carried out on an automated analyzer (Free Carpe Diem, Diacron International, Grosseto, Italy), using a commercial kit (Diacron International) according to the manufacturer’s instructions, as previously reported (Barrea et al., 2018c) The LP-CHOLOX test evaluates a class of hydroperoxides derived from the lipid peroxidation, which are mainly represented by oxidized cholesterol. The peroxides are able to promote the oxidation of the ferrous iron (Fe^2+^) to ferric iron (Fe^3+^). The LP-CHOLOX test is based on the spectrophotometric measurement (at 505 nm) of the colored complex developed by the binding between the Fe^3+^ and the thiocyanate. The absorbance values are directly proportional to the lipoperoxides concentrations, and the values are related to specific standard solution (400 μEq/L). Results are expressed in μEq/L, and reference values are: normal, ≤599 μEq/L; slight alteration, from 600 to 799 μEq/L; moderate alteration, from 800 to 999 μEq/L; strong alteration, ≥1000 μEq/L (Macri et al., 2015; Mancini et al., 2017).

### Preparation of Serum Samples and Standards

For the quantification of circulating levels of TMAO from serum samples, 80 μl of serum were added to 160 μl methanol, vortexed for 30 s and centrifuged at 14,000 rpm for 10 min (4°C), as reported in our previous studies (Barrea et al., 2018a,b; Annunziata et al., 2019). An aliquot of supernatant was transferred to HPLC vial for the LC/MS analysis. TMAO standard solution was freshly prepared by dissolving TMAO analytical standard (Sigma-Aldrich, Milan, Italy) in methanol to obtain a 1.0 mg/mL stock solution. Serial dilutions were prepared in phosphate buffered saline (PBS, pH 7.4, Sigma-Aldrich, Milan, Italy) from the stock solution, to create standard dilutions at concentrations of 0.033, 0.333, 0.667, 3.333, 6.667, 13.333, and 26.667 μM.

### Equipment and HPLC-MS Conditions

**FIGURE 1:**
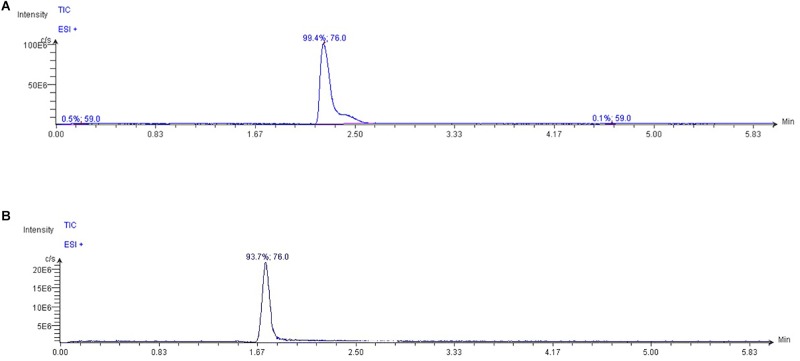
Typical extracted ion chromatograms from LC/MS analysis of **(A)** TMAO and **(B)** glycine standard dilution (ion extracted at m/z = 76.0 in positive mode). As glycine is present in plasma and having both the same TMAO molecular weight and ability to be ionized in positive mode, the two different retention times allow to discriminate TMAO in biological fluid from other compounds with similar chemical features.

The High-Performance Liquid Chromatography (HPLC) system Jasco Extrema LC-4000 system (Jasco Inc., Easton, MD, United States) was coupled to a single quadrupole mass spectrometer (Advion ExpressIon^L^ CMS, Advion Inc., Ithaca, NY, United States) equipped with an Electrospray ionization (ESI) source, operating in positive ion mode. The chromatographic separation was performed with a Luna HILIC column (150 mm × 3 mm, 5 μm particles) in combination with a guard column (HILIC), both supplied by Phenomenex (Torrance, CA, United States). The oven temperature was set at 60°C. Mobile phase A was 0.15% formic acid and 10 mM ammonium acetate in water; mobile phase B was 100% methanol (LC/MS grade). According to Beale and Airs (2016), isocratically run was performed at a flow rate of 0.35 ml min^-1^ for 6 min, in the ratio 80:20 (A:B), with an injection volume 10 μL. The capillary temperature was set at 300°C; capillary voltage + 150 V; source voltage offset + 25 V; source voltage span + 70 V; gas source temperature 350°C; ESI voltage 3500 V. Data were acquired in SIM mode. TMAO was detected at m/z 76 (Figure 1); its fragments were monitored at m/z 58 and 59. Mass spectra were recorded from m/z = 50–500.

### Calibration and Linearity

**FIGURE 2:**
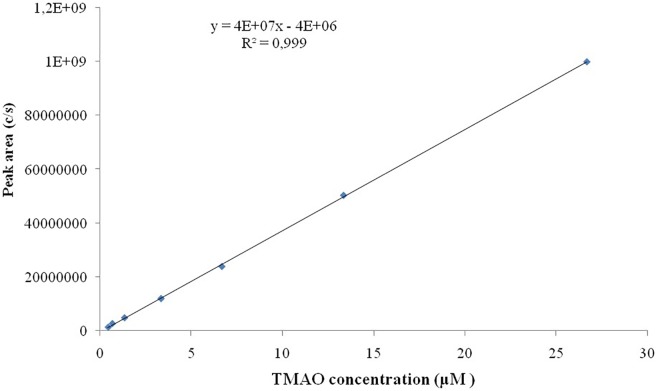
Calibration curve of TMAO over the concentration range of 0.033–26.667 μM.

**FIGURE 3:**
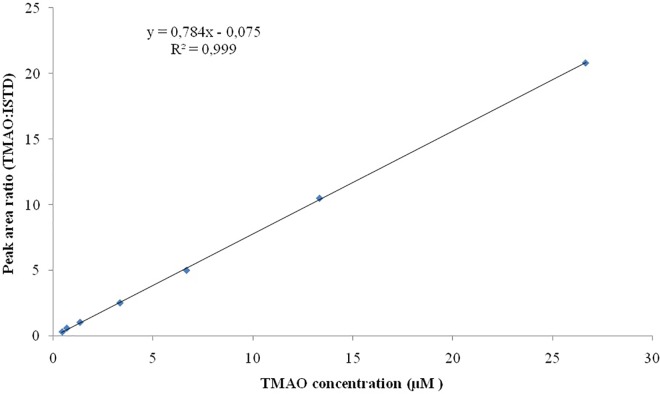
Linearity for TMAO over the concentration range of 0.033–26.667 μM. All the analysis are run in duplicate.

Seven TMAO dilutions (0.033, 0.333, 0.667, 3.333, 6.667, 13.333, and 26.667 μM) were used to construct the calibration curve (Figure 2). Each calibration level was run in duplicate for three consecutive days, except for the lower limit of quantification, which was run in triplicate. To evaluate the linearity of the studies performed, the plots obtained from the injection of each calibration levels versus peak area ratio (analyte peak area/internal standard peak area) were plotted with a correlation coefficient of *R*^2^ 0.999 (Figure 3).

### Accuracy, Precision, Recovery, and Sensitivity

The limits of detection (LODs) and the limits of quantification (LOQs) were studied to define the sensitivity of the performed analysis. LOD, is commonly defined as the lowest detectable concentration of analyte that the system can reliably differentiate from the baseline level (with a ratio of analytics signal/baseline noise = 3) (Le et al., 2018). While LOQ is defined as the lowest quantifiable amounts of analyte that can be measured with a standard level of confidence and it is generally elaborated using the ratio Signal/Noise = 10 (Le et al., 2018). Precision and accuracy of the methods were assessed by intra-day and inter-day analysis of selected standard dilutions. Standard dilutions were injected six times in the same day (inter-days), and the same dilutions were injected a single time a day for 10 consecutive days (intra-days). The precision of the method was determined by the calculation of coefficient of variation (% C.V.) relative to the comparison of the peak area deriving from latter experiments. While the accuracy was expressed as bias % and deriving from the calculation of mean concentration relative to the nominal concentration of each standard dilution analyzed. Recovery was analyzed by spiked a pooled (*n* = 3) serum sample (100 μL) with 10 μL of standard solution at three different concentrations (0.333, 3.333, and 13.333 μM) of TMAO. The recovery was calculated by comparing the nominal concentration to the experimental concentration.

### Statistics

Unless otherwise stated, all the experimental results were expressed as mean ± standard deviation (SD) of three determinations. Statistical analysis of data was performed by the Student’s *t*-test or two-way ANOVA (SPSS 13.0) followed by the Tukey–Kramer multiple comparison test to evaluate significant differences between a pair of means. *P*-values less than 0.05 were regarded as significant. The chi-square (χ2) test was used to determine the significance of differences in frequency distributions.

## Results

### Effect of Taurisolo^®^ With or Without Pectin in Reducing ox-LDL and TMAO Serum Levels

**FIGURE 4:**
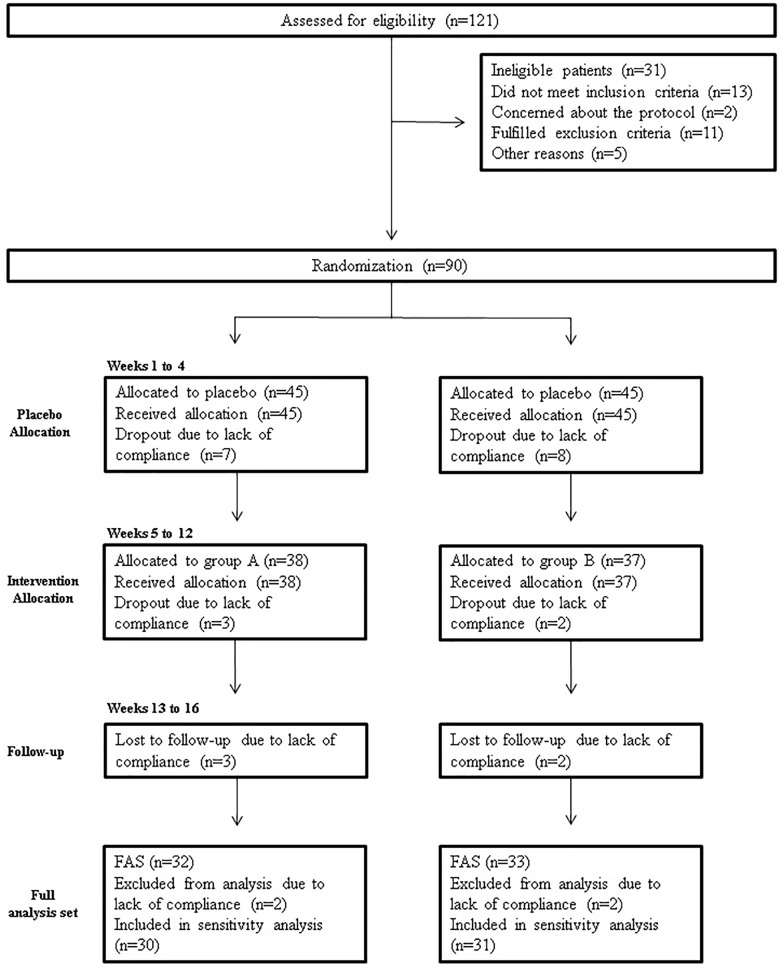
Study flowchart, according to the consolidated standards of reporting trials (CONSORT). The diagram shows enrolment and primary efficacy endpoints based on patients’ diaries, from pre-screening to data collection; and the extent of exclusions, loss to follow-up, and completeness of diary documentation available across the entire trial period. FAS, Full analysis set.

A total of 121 subjects were screened for eligibility; 90 were randomized, while 31 (25.6%) did not pass the screening stage. Selected patients were randomized into groups A and B. The study flow chart according to the CONSORT PRO reporting guideline (Calvert et al., 2013) is represented in Figure 4.

**FIGURE 5:**
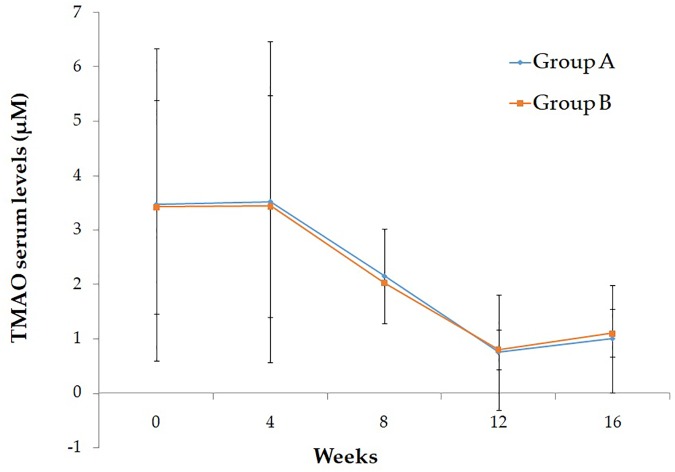
Graphical representation of the TMAO serum levels at the different time points.

Baseline characteristics of study participant are reported in Table 1. As shown, no significant differences were evident between the two intervention groups. After the run-in period, during which participants were given placebo, no differences have been observed in the ox-LDL and TMAO serum levels. In Table 2 are reported the effect of Taurisolo^®^ or Taurisolo^®^+pectin on ox-LDL and TMAO serum levels. In both intervention groups TMAO serum levels significantly decreased (ox-LDL: -76.06% *p* = 0.03 and -74.02% *p* = 0.02, group A and group B, respectively; TMAO: -78.58% *p* = 0.006 and -76.76% *p* = 0.001, group A and group B, respectively). No significant differences have been reported between the two interventions after the 8-week treatment period (ox-LDL: *p* = 0.915 and TMAO: *p* = 0.897, group A vs. group B). After the 4-week follow-up period, the ox-LDL and TMAO serum levels not significantly increased in both groups (ox-LDL: 23.85 and 25.83%; TMAO: 33.77 and 37.60%, group A and group B, respectively). In Figure 5 are graphically represented the TMAO serum levels at the different time points.

**Table 1:** Baseline characteristics of study participants.

Parameters	Group A	Group B	*p*-Value
Gender [male (%)]	57.14	55.00	χ^2^ = 0.019, *p* = 0.890
Age (years)^∗^	65.14 ± 10.46	64.50 ± 11.84	0.855
Physical activity [yes (%)]	14.29	5.00	χ^2^ = 1.003, *p* = 0.316
Weight (kg)^∗^	83.25 ± 12.54	83.48 ± 13.53	0.956
Height (m)^∗^	1.64 ± 0.10	1.63 ± 0.11	0.732
BMI (kg/m^2^)^∗^	31.16 ± 4.74	31.54 ± 3.55	0.776
WC (cm)^∗^	107.08 ± 12.37	106.25 ± 9.11	0.812
HC (cm)^∗^	113.05 ± 13.78	110.55 ± 7.75	0.484
WHR^∗^	0.95 ± 0.11	0.96 ± 0.07	0.604
Diabetes mellitus [yes (%)]	47.62	45.00	χ^2^ = 0.028, *p* = 0.866
Hypertension [yes (%)]	42.86	30.00	χ^2^ = 0.729, *p* = 0.393
Hypercholesterolemia [yes (%)]	66.47	60.00	χ^2^ = 0.196, *p* = 0.658
Hypertriglyceridemia [yes (%)]	4.76	10.00	χ^2^ = 0.414, *p* = 0.520
TMAO (μM)^∗^	3.52 ± 2.95	3.44 ± 2.04	0.926
ox-LDL (μEq/L)	1041.21 ± 406.76	941.54 ± 316.01	0.754

*No significant differences were evident between the two groups. ^∗^Values are expressed as mean ± SD; statistical significance is calculated by Student’s t-test. BMI, body mass index; WC, waist circumference; HC, hip circumference; WHR, waist-to-hip ratio; SD, standard deviation; TMAO, trimethylamine N-oxide.*

**Table 2:** ox-LDL and TMAO serum levels of study participants before and after 2-month treatment.

		ox-LDL serum levels (μEq/L)			Δ (%)	*p*-value
	Run-in (placebo)	Before treatment	After treatment	Follow-up		
Group A	1102.33 ± 398.43	1041.21 ± 406.76	249.2 ± 47.01	308.64 ± 52.57	-76.06	0.03
Group B	822.99 ± 370.94	941.54 ± 316.01	244.6 ± 52.4^∗^	307.76 ± 100.77	-74.02	0.02

		**TMAO serum levels (μM)**			**Δ (%)**	***p*-value**
	**Run-in (placebo)**	**Before treatment**	**After treatment**	**Follow-up**		

Group A	3.47 ± 2.87	3.52 ± 2.95	0.75 ± 1.06	1.00 ± 0.99	-78.58	0.006
Group B	3.42 ± 1.97	3.44 ± 2.04	0.80 ± 0.37^#^	1.10 ± 0.44	-76.76	0.001

*Value are expressed as mean ± SD of three replicates. Decrement percentage between ox-LDL and TMAO serum levels before and after 8-week treatment was shown. Statistical significance is calculated by Student’s t-test. ^∗^P = 0.915, group A vs. group B (after treatment); ^#^P = 0.897, group A vs. group B (after treatment). SD, standard deviation; TMAO, trimethylamine N-oxide.*

### Precision and Accuracy

The intra-day and inter-day accuracy (% bias) and precision (% C.V.) were determined at the concentrations of 0.033, 0.333, 0.667, 3.333, 6.667, 13.333, and 26.667 μM (Table 3). As expected, the higher % C.V. was measured at lower concertation tested (0.0025 ppm) with intraday and interday % C.V. of 12.12 and 8.25%, respectively. The same was for the accuracy, where the lower values of % bias were obtained at the lower concentration tested, with % bias of -5.82% (intra-day) and of -1.41% (inter-day). Generally, we have found that % C.V. values ranged from 12.12 to 3.92% and from 8.25 to 1.07% for intraday and interday precision, respectively; the % bias ranged from -5.52 to 0.5% for the estimation of intraday accuracy and from -1.42 to 3.08% for the evaluation of interday accuracy. TMAO recoveries from serum ranged from 99 to 97% depending on the three different standard dilutions used (Table 4).

**Table 3:** Intra-day and inter-day precision and accuracy of the LC/MS method.

TMAO concentration tested (μM)	Intra-day precision (% C.V. *n* = 6)	Intra-day accuracy (% bias *n* = 6)	Inter-day precision (% C.V. *n* = 6)	Inter-day Accuracy (% bias *n* = 6)
0.033	8.25	-5.52	12.12	-1.42
0.333	8.12	-3.52	10.5	1.02
6.667	1.78	0.89	7.20	1.42
3.333	1.54	2.6	4.89	0.85
6.667	1.10	0.62	5.20	0.79
13.333	1.52	0.66	3.95	0.65
26.667	2.75	0.45	4.68	-0.69

**Table 4:** Recovery of TMAO in pooled patient serum (*n* = 3).

Baseline TMAO concentration in polled t0 patients’ serum	Spiked concentration (μM)	Measured	% Recovery
5.347 ± 0.003	0.333	5.679 ± 0.360	99.99
	3.333	8.532 ± 0.063	98.30
	13.333	18.121 ± 1.083	97.01

### Linearity and Sensitivity

Linearity studies were conducted by the preparation of calibration curves on a wide range of analytical standard concentrations (seven dilutions ranging from 0.033 to 26.667 μM). All the analyses were conducted in triplicate, the ratio of standards concretions versus peak area ratio (analyte peak area/internal standard peak area) was plotted with a correlation coefficient of *R*^2^ 0.999. Our results have indicated the LOD is 2 ng/mL while LOQ is of 6 ng/mL.

## Discussion

In the present study, we demonstrated the ox-LDL- and TMAO-reducing effect of a novel nutraceutical formulation based on grape polyphenolic extract (Taurisolo^®^, treatment group A) in a cohort of overweight/obese subjects. To the best of our knowledge, no previous study has investigated the effect of grape polyphenols in overweight/obese. The first result we observed was a significant decrease of the ox-LDL and TMAO serum levels after 8-week treatment. Additionally, considering the well-established role of the gut microbiota in the regulation of the TMAO serum levels, we also tested the effect of Taurisolo^®^+pectin (treatment group B), which microbiota-modulating effect has been previously demonstrated (Licht et al., 2010; Jiang et al., 2016). We found no significant differences between the after-treatment ox-LDL and TMAO serum levels in the two intervention groups, suggesting that the addition of pectin does not represent a value added for the observed effect of our nutraceutical formulation and, probably, the main role is played by grape polyphenols.

In a previous study, we demonstrated the TMAO-reducing effect of Taurisolo^®^ in young healthy subjects, proposing a potential mechanism of action for the effect of polyphenols in reducing TMAO levels, based on the ability of polyphenols to donate electrons to TMAO, which act as an electron acceptor, resulting in reducing TMAO to TMA (Annunziata et al., 2019). These redox reactions would occur at blood level. The hypothesis of an antioxidant effect of Taurisolo^®^ polyphenols exerted at serum levels is corroborated by the observed reduction of ox-LDL serum levels, as a circulating oxidative stress biomarker, appearing of interest for the clinical management of the CVD risk.

As TMAO is a highly oxidant and reactive molecule, the link connecting oxidative status and cardiovascular risk is intuitive (Pignatelli et al., 2018). The term oxidative stress describes the unbalance between production of reactive oxygen species (ROS) and antioxidant clearance (Sies, 2015). Unfavorable conditions, such as obesity, diabetes, hypertension or dyslipidemia, have been recognized as causes of increased ROS levels (Violi et al., 2017b; Garramone et al., 2018). Evidences supported the strong relationship between oxidative stress and CVD, including atherosclerosis (Steinberg et al., 1989; Steinberg, 2009; Steinberg and Witztum, 2010). During the atherosclerotic process, plaque formation is caused by cytokines and ROS-induced molecular changes, including oxidization of LDL, which accumulate in the subendothelium. ox-LDL, in turn, induces the production of adhesion molecules, resulting in monocytes and T-cells recruitment, and subsequent release of pro-inflammatory cytokines and ROS. This process is responsible for apoptosis and foam cell formation, resulting in production of the atherosclerotic plaque (Violi et al., 2017a). Notably, TMAO enhances the oxLDL-induced expression of scavenger receptor CD36 in macrophages, resulting in increased accumulation of lipids and formation of foam cells (Wang et al., 2011, 2015; Geng et al., 2018).

*In vitro* studies elucidated the mechanisms by which polyphenols reduce oxidizability of LDL. In particular, it has been reported that polyphenols are able to upregulate the autophagic pathways (Chen et al., 2017) and catechins are incorporated into the LDL particles (Suzuki-Sugihara et al., 2016), resulting in reduced oxidizability of LDL. Beside the highly reactivity of catechol moiety in preventing oxidative processes (Hirano et al., 2001), it has been reported that catechins are able to exert radical trapping effects and act as hydrogen donors to α-tocopherol radicals, resulting in prevention of LDL oxidation (Zhu et al., 1999). Interestingly, according to our previous analyses, Taurisolo^®^ contains both catechin and epicatechin, on average 10.87 and 8.86 mg/g, respectively (Annunziata et al., 2019). Additionally, *in vitro* studies investigating the effects of red wine polyphenols, conducted in order to elucidate the mechanisms at the base of the well-known French paradox, reported the ability of this class of polyphenols to inhibit the oxidization of LDL catalyzed by copper (Frankel et al., 1993).

The observed antioxidant effect of Taurisolo^®^, which may potentially occur at serum level, are justified by our previous Taurisolo^®^ polyphenols bioaccessibility and bioavailability studies. Our supplement, indeed, consists of acid-resistant capsules, which strongly reduce the loss of polyphenols due to the biochemical changes occurring during the gastrointestinal digestion, resulting in increased intestinal bioaccessibility (Annunziata et al., 2018, 2019). Furthermore, the spray drying-based microencapsulation with maltodextrins significantly increases the water solubility of polyphenols, enhancing their intestinal absorption and bioavailability (Annunziata et al., 2019). Overall these evidences, suggest that, although several studies recognized the microbiota modulation as the main approach to reduce and/or maintain the blood levels of TMAO, novel interpretations might be proposed, and the role of nutraceutical is emerging.

Currently, a large number of studies is focusing on the relationship between TMAO and several diseases, mainly related to cardiovascular risk, suggesting the importance to monitor this blood parameter in the clinical practice. Several methods have been developed, and among these NMR and MS are emerging (Cheng et al., 2017; Albert and Tang, 2018). Beside the pros and cons identified for these two methods, MS may be considered more useful and practice not only in the research field but also in the clinical diagnostic.

The MS-based method herein used was previously developed and by Beale and Airs (2016) for evaluation of TMAO levels in seawater. We optimized the method for detection of this metabolite in serum and validated it. Validation was performed not for a comparison with other previously published results, but to confirm the validity of our experiments. Our data suggest that our method has good precision and accuracy, with a % C.V. ranging from 12.12 to 3.92% and from 8.25 to -1.07% for intraday and interday analyses, respectively; additionally, % bias ranged from -5.81 to 0.5% and from -1.41 to -3.08% for interday and intraday analyses, respectively. LOD and LOQ values were 2 and 6 ng/ml, respectively. These values are closely in line with previous findings, such as reported by Gibb and Hatton (2004) (LOD: 2 nM), Beale and Airs (2016) (LOD: 3.9 nM), Heaney et al. (2016) (LOD: 5 nM), and Le et al. (2018) (LOQ: 6 ng/mL). Furthermore, LODs and LOQs values are largely below the concentrations detected for serum samples in our laboratory.

In summary, in the present study, we demonstrated the efficacy of Taurisolo^®^ in reducing ox-LDL and TMAO serum levels in overweight/obese subjects. This main finding appears relevant in consideration of the well-established role of overweight/obesity as risk factors for CVD, and the association with higher TMAO serum levels (Barrea et al., 2018a). We found that the addition of pectin did not improve the TMAO-reducing effect of Taurisolo^®^, suggesting that polyphenols may be the main actors. According to the available literature, however, polyphenols might play their TMAO-reducing effect both remodeling the gut microbiota (Lee et al., 2006; Selma et al., 2009) and exerting the antioxidant activity at serum level (Annunziata et al., 2019). The results herein presented appear interesting for both novelty and applicability, highlighting the potential of nutraceutical in management or prevention of CVD, mainly considering that TMAO is currently under the spotlight for its role as prognostic biomarker of cardiovascular risk. Interestingly, our results may provide a novel interpretation for the well-known cardioprotective effects of grape polyphenols, leading physician, in general, and cardiologist, specifically, to consider the use of grape polyphenolic extract in management of high risk subjects as *add-on* therapy. Furthermore, herein we presented a useful, rapid, versatile and valid method for detection of TMAO concentration in serum, which would be considered for further applications in the clinical diagnostic and nutraceutical research. This study, however, is not without limitations. Firstly, specific cardiovascular outcomes have not been monitored; this is due to the main aims of this study, including the evaluation of the TMAO-reducing effect of our supplement and validation of the method performed in our laboratory. Moreover, the study of the microbiota would have allowed to establish whether the nutraceutical formulations we proposed determined a modulation of the microbiota in favor of the reduction of serum TMAO levels. On the other hand, our data may be useful for physicians, informing them about a novel nutraceutical remedy for the treatment of high cardiovascular risk-subjects.

## Data Availability

All datasets generated for this study are included in the manuscript and/or the supplementary files.

## Ethics Statement

The study was conducted in accordance with the Declaration of Helsinki, and the protocol was approved by the Ethics Committee of AO Rummo Hospital (Benevento, Italy) (protocol 106 no. 123512 of 18/06/2018). This study is listed on the ISRCTN registry (www.isrctn.com) with ID ISRCTN10794277 (doi: 10.1186/ISRCTN10794277).

## Author Contributions

GA and MM did conception and design, analyzed and interpreted the data, and drafted the manuscript. GA, MM, CS, RC, and VN carried out the experiments and acquired the data. SH, GT, and EN supervised the manuscript. All authors critically revised the manuscript.

## Conflict of Interest Statement

The authors declare that the research was conducted in the absence of any commercial or financial relationships that could be construed as a potential conflict of interest.
